# Investigating Intraspecific Attacks in Brown Bears (*Ursus arctos*) Using a Forensic Approach: Evidence from Northern Italy

**DOI:** 10.3390/ani16071119

**Published:** 2026-04-06

**Authors:** Cristina Marchetti, Roberto Guadagnini, Rosanna Di Lecce, Luca Ferrari, Gennaro Carrozzo, Sofia Guadagnini, Andrea Mazzatenta

**Affiliations:** 1 Department of Veterinary Science, University of Parma, 43126 Parma, Italy; rosanna.dilecce@unipr.it (R.D.L.); luca.ferrari@unipr.it (L.F.); 2 Neurophysiology of Olfaction and Chemoreception Laboratory, Science Department, “G. d’Annunzio” University of Chieti-Pescara, 66013 Chieti, Italy; 3 GPG Wildlife Project, 38017 Trento, Italy; roberto@zoolife.it (R.G.); gennaro.carrozzo@gmail.com (G.C.); sofia.guadagnini@gmail.com (S.G.)

**Keywords:** brown bear, intraspecific aggression, forensic genetics, forensic odontology, inter-canine distance (I-CD), bite mark analysis, international classification of diseases 11th revision (ICD-11), ecophysiology

## Abstract

Fatal intraspecific aggression in brown bears (*Ursus arctos*) remains poorly documented, yet elucidating its dynamics is critical in order to understand species’ physiology, informing management strategies, and advancing wildlife forensic science applications, which are useful in cases where a natural or illegal cause of death needs to be discerned. In this study, we reported four confirmed cases of lethal aggression (two yearlings and two adults) in the Italian Alps. Comprehensive autopsies were performed to characterize lesion patterns and infer the aggressor identity. Claw-induced lacerations, bite marks and the aspect of hemorrhages suggested the attack sequence. Aggressor identity was investigated by using forensic odontology through inter-canine distance (I-CD) and genetic analysis of peri-lesional saliva. I-CD allowed us to plausibly hypothesize the aggressor’s species and, in the cases where it was possible, to classify the sex and/or age group of the aggressors. While genetic analysis allowed the identification of the four brown bear victims, it did not provide informative results on the aggressors. The cause and manner of death were coded according to international criteria (International Classification of Diseases 11th Revision [ICD-11], WHO). Adult fatalities, supported by gastric content analysis, reflect trophic competition regardless of the mating context and highlight the role of anthropogenic food sources in conflict emergence. These findings underscore the value of integrated approaches in wildlife investigations and provide new insights into ecophysiological factors driving lethal intraspecific aggression.

## Simple Summary

Bears predominantly exhibit solitary behavior, minimizing intraspecific and interspecific interactions. However, during the mating season or periods of resource aggregation, spatial overlaps increase, thus forcing temporary social tolerance. These interactions can escalate into aggressive behavior, sometimes leading to serious or fatal injuries. Such events are rarely documented, which prompted us to investigate how these encounters occur and what driving factors are involved. We examined four mortality cases involving brown bears from the Italian Alps. The post-mortem examination studied and described the lesional patterns and sequence of the attack. The stomach content analysis revealed the presence of anthropogenic food items that may have attracted bears to overlapping areas with humans, thus increasing the conflict risk. Understanding the motivations of a bear attack is crucial to avoid situations of danger and minimize the chances of intraspecific and interspecific conflicts. The correct use of forensic tools can help us understand the dynamics and, consequently, increase the compatibility between humans and wildlife.

## 1. Introduction

In the Italian Alps, bears belong to the nominal subspecies *Ursus arctos arctos*, reintroduced through the Life Ursus Project [1], started in 1998 by the Italian Ministry of the Environment and the European Community to restore the population in the Adamello Brenta Natural Park, in the Trentino region, Italy.

Bears are predominantly solitary, minimizing intraspecific encounters. Yet, during the mating season or in contexts of resource aggregation, either natural [2,3] or anthropogenic, such as access to human-derived food sources, spatial overlaps increase, imposing temporary social tolerance. These interactions, often marked by chemical communication and competition for resources, can escalate into aggressive conflicts, leaving evidence, such as animal corpses from which to infer information that can indicate the ecophysiological mechanisms that shape population dynamics, inform conservation strategies, and help recognize the species responsible for an attack.

The main characteristic of the species is an opportunistic omnivore behavior, with a predominantly vegetarian diet supplemented by insects, honey, and occasional scavenging of ungulates, particularly in spring [4]; brown bears exhibit marked sexual dimorphism in body size which increases with age [5,6,7,8,9]. Body weight shows seasonal fluctuations, with post-hibernation declines, despite access to anthropogenic food sources such as ungulate artificial feeding sites set up by hunters [10], a practice that increases intra- and inter-species interactions and influences population dynamics through higher reproductive rates and greater cubs’ survival [11,12,13]. According to several studies, the diet of brown bears includes approximately 10% wild ungulates, derived both from scavenging, such as the use of wolf-killed preyed animals, or their remains, in multi-predator systems [14], and from active predation, with peaks in late spring and a preference for neonates and large species [15,16].

In brown bear populations, lethal intraspecific interactions may occur, involving cubs, yearlings, subadults, and occasionally older individuals. Infanticide is well-documented as a male reproductive strategy in some mammals and termed sexually selected infanticide (SSI) [3].

This strategy is common in polygamous species with prolonged maternal care and lactational anestrus. Females with cubs often adopt avoidance strategies, altering habitat selection to reduce the SSI risk [6] and may exploit anthropogenic areas as refuges against male aggression [17]. In addition to SSI, lethal interactions have also been documented among independent subadults. Balseiro and co-workers in 2020 reported four fatalities out of 25 individuals in the Cantabrian Mountains (northern Spain) following intraspecific conflicts [18]. Similarly, a Swedish study described 16 cases of bear-caused mortality, of which 15 were classified as infanticide, while only one involved an adult female who was killed along with her cub [19].

The ethological drivers of intraspecific attacks unrelated to reproductive strategies are still poorly understood [3]. Similarly, interactions during specific behaviors such as resting, foraging, and reproduction remain largely unexplored [14].

Some authors attribute these events to age-related dominance behaviors. Swenson and co-workers observed that the number of victims from such aggression is relatively low and hypothesized that the removal of subadult males by dominant adults may contribute to population regulation [3]. Adult males, and occasionally females [20], are typically the aggressors, and when attacks involve two adults, they most occur in contexts of food competition [21].

Chemical communication, the most ancient form of intraspecific and interspecific non-verbal signaling, plays a pivotal role in diverse biological processes and cannot be disregarded in this context [22]. Recent studies have explored chemical communication in bears, highlighting the complexity of an “evanescent bouquet” of emitted molecules [23]. Interestingly, exposure to fear-related chemo-signals, such as sweat from fearful individuals, can elevate physiological responses in predators [24,25]; consequently, survival-related clues enhance specific reactions. Mammalian predators are attracted by specific chemical signals (chemoattractants) released by prey or present in the environment, which indicate food availability, or conversely the presence of conspecifics, or territorial marking. Further, the odor of mammalian blood is a strong attractant for apex predators. This odor is capable of eliciting attraction responses like those induced by whole blood odor in large carnivores [26].

In the scientific literature, descriptions of lesion patterns or attack dynamics are rare; published studies focus mainly on statistical data, such as the number of livestock attacks [27], preventive measures such as barriers [28], or hive protection methods [29], leaving a significant gap in the characterization of injuries.

The present study describes the injuries caused by a bear attack and the sequence of the attack inferred from the different patterns of the lacerations found, accompanied by various degrees and extensions of perilesional hemorrhages. The aim is to provide a guidance for the recognition of lesions in future similar cases and to focus on what, with a high degree of scientific certainty, represents the motivation for the attack (contention of trophic resources). In particular, the bite signs were analyzed as a possible tool for recognizing the aggressor’s species and, in the cases in which it was possible, to recognize the sex and/or age group of the aggressor. The merits and limitations of the results of forensic genetic investigations are also highlighted.

It is believed that the information obtained should be taken seriously to prevent accidents between bears and animals of other species, including humans, and to promote awareness and responsibility in the relationship with wildlife.

## 2. Materials and Methods

### 2.1. Study Area and Cases

**Figure 1:**
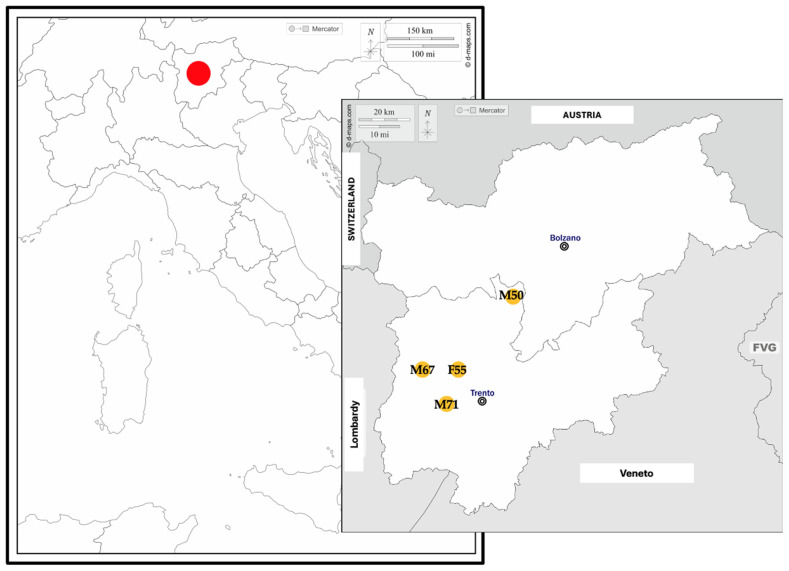
The study area is the Adamello Brenta Natural Park, a protected area located in the Trentino region, Italy. It covers 620.5 km^2^ and ranges from 477 to 3558 m above sea level. Established in 1967, the Park encompasses two major mountain massifs: the Brenta Dolomites, designated as a UNESCO World Heritage Site, and the Adamello/Presanella Alps. It is also recognized as a UNESCO Global Geopark (UGGp) due to its 61 geosites. Conservation and management activities are supported by the European Agricultural Fund for Rural Development (EAFRD), the Italian Government, and the Autonomous Province of Trento (PAT). Source: [30]. In the present study, the brown bears found were: M50, an adult male aged 5 years and 8 months; M71, an adult male aged 2 years and 4 months; M67, a male yearling aged 15 months; F55, a female yearling aged 16 months. The cartographic image was assembled and adapted from d-maps.com: https://www.d-maps.com/carte.php?num_car=4831&lang=it (Italy) (accessed on 29 March 2026) and https://d-maps.com/carte.php?num_car=8338&lang=it (Trentino-Alto Adige region, Italy) (accessed on 29 March 2026). In the cartographic image of Italy, the red spot indicates the Trentino-Alto Adige region. In the cartographic image of the Trentino-Alto Adige region, the orange spots indicate the respective locations where the brown bear corpses were found. FVG: Friuli-Venezia Giulia region.

The study was conducted in the forests of the Adamello Brenta Natural Park, located in the Trentino region of the Italian Alps (Figure 1).

In spring 2020, the corpses of two brown bear yearlings were recovered in separate events: a female (F55), 16 months old, was found on 20 May 2020, at UTM coordinates (Zone 32T, WGS84): 645,996 E, 5,106,666 N—Val d’Ambiez, San Lorenzo Dorsino, Autonomous Province of Trento (PAT), Italy; a male (M67), 15 months old, was found on 1 April 2020, at UTM coordinates (Zone 32T, WGS84): 634,251 E, 5,109,236 N—Pler, Strembo, PAT, Italy.

In 2021, two independent cases of suspected fatal intraspecific aggression were documented: one in spring and one in late summer. The first case involved a young adult male bear at the time identified with the code OM20210506TN and subsequently genetically identified as M71, 2 years and 4 months old, recovered on 6 May 2021, in a wooded area at Viote del Bondone, PAT, Italy (UTM Zone 32T, WGS84: 657,649 E, 5,098,099 N).

The second case, recorded on 15 September 2021, concerned an adult male bear at the time identified with the code OM1592021TN and subsequently genetically identified as M50, 5 years and 8 months old, found in a muddy puddle within a forest clearing at Sasso Magno, Cles Mountain, PAT, Italy (UTM Zone 32T, WGS84: 651,024 E, 5,132,560 N).

Age was determined based on genetic testing. The age of brown bear M71 was estimated through morphometric findings, verified through histological testing, and then determined through genetic testing.

### 2.2. On-Site Inspection

On-site inspections were carried out by the Provincial Forestry Corps of the Trentino region. Cadavers were photographed in situ, georeferenced using handheld GPS devices (accuracy ≈ 5 m). Cadavers were then transferred to the Large Carnivore Veterinary Unit for CT-based diagnostics and subsequently subjected to autopsy and auxiliary tests.

### 2.3. CT-Based Diagnostics

Computed tomography (CT) was performed to identify skeletal fractures by using an Aquilion Lightning™ CT scan, 16-row, 32-slice helical system (Canon Medical Systems, Rome, Italy). Diagnostic imaging was employed as part of the post-mortem investigation in brown bear M71 to facilitate the three-dimensional (3D) reconstruction of the skeletal architecture and trauma distribution, aiming to elucidate the dynamics and severity of the agonistic interaction. Post-mortem CT provided high-resolution volumetric datasets enabling precise visualization of osseous displacement and fracture morphology.

### 2.4. Post-Mortem Investigation

Each cadaver underwent a thorough external examination at the facility made available by the authorities, during which the injuries, bite marks, and claw traces were studied and documented using metric scales and high-resolution photography. Morphometric measurements, including weight, body length (occiput-to-tail base), and neck circumference, were recorded with calibrated instruments. Inter-canine distance (I-CD) obtained by clearly identifiable bite mark measurements was analyzed to guide investigations towards the identification of the sex and/or age group of the aggressor. In addition to the existing literature [31], local reference I-CD values were obtained from three adult female brown bears (body masses/age: F9, 150 kg, 8 years and 9 months old; F36, about 80 kg, 6 years and 8 months old; F43, 140 kg, 4 years and 8 months old) that died due to unrelated causes, respectively vehicular collision, gunshot injury, and trapping incident.

Autopsies were performed following a stepwise, layer-by-layer dissection approach to document all anatomical structures and lesions. Cadavers were skinned to allow the study of the skin and subcutaneous tissues. A systematic evaluation of hemorrhages and trauma patterns was performed. Internal organs were examined. The thymus and the only testis found in bear M71 were collected and analyzed histologically.

During the autopsy, gross examination of the cavitary organs was conducted and samples suitable for animal health checks were collected.

### 2.5. Genetics

During the post-mortem examination, biological samples (*papillae labiales*) were collected from all four brown bears under study for the identification of each subject. In order to identify the aggressor through salivary DNA testing, swabs were taken from bite wounds, where present, that showed obvious vital signs (hemorrhagic extravasation). To collect biological material from the aggressor, which could have been transferred during the combat between the two adults, swabs were obtained by rubbing the surface of the teeth, and the distal phalanges (*os unguiculare*) were removed. The swabs were air-dried and stored in paper bags, while the distal phalanges were stored in sterile plastic containers. All sample containers were labeled with the sample code and the location of the sampling point on the corpse and addressed to the Edmund Mach Foundation (FEM) (San Michele all’Adige, Trento, Italy) for genetic identification and age determination [32]. FEM, particularly through its Conservation Genetics Research Unit (CONGEN) and Animal Ecology Unit is involved in the monitoring and study of the brown bear population in the Italian Alps. Age determination was carried out based on data from non-invasive genetic sampling, visual observations, and phylogenetic/ecological information from the individuals.

### 2.6. Histology

The histological examination was performed on the thymus and testis of bear M71 to confirm the age range and know the reproductive phase suggested by the morphological data. Tissue specimens for histological analysis were fixed in 10% neutral buffered formalin, embedded in paraffin, sectioned at 5 μm, and stained with hematoxylin and eosin (H&E). Slides were examined by using a Nikon Eclipse E800 microscope and digital images were acquired by using a Digital Sight DS-Fi1 camera (Nikon Corporation, Tokyo, Japan).

### 2.7. Classification of Cause and Manner of Death

The classification of the cause and manner of death was coded according to international criteria (International Classification of Diseases 11th Revision [ICD]-11, the global standard for diagnostic health information; World Health Organization, WHO) [33] as recommended by Marchetti and colleagues [34].

### 2.8. Behavioral Assessment

The behavioral assessment aimed at elucidating the underlying drivers for intraspecific lethal aggression. The study relied on a thorough assessment of the technical findings from the veterinary investigation. This included detailed observations at corpse finding locations, autopsy findings, and ancillary examinations. These data were integrated with species-specific ethological profiles to reconstruct the behavioral dynamics underlying the event. Particular attention was given to indicators such as feeding patterns, wound distribution, and evidence of a territorial or predatory behavior. The analysis aimed to distinguish between natural predation, scavenging, and intraspecific aggression, providing a contextually informed interpretation of the observed injuries and environmental traces.

## 3. Results

### 3.1. Geographical Map of the Findings

All brown bear corpses were recovered at geo-referenced locations within the Adamello Brenta Natural Park, Trentino region, Italy, as shown in Figure 1 (M71: young-adult male brown bear; M50: adult male brown bear; M67: yearling male brown bear; F55: yearling female brown bear).

### 3.2. On-Site Inspection

**Figure 2:**
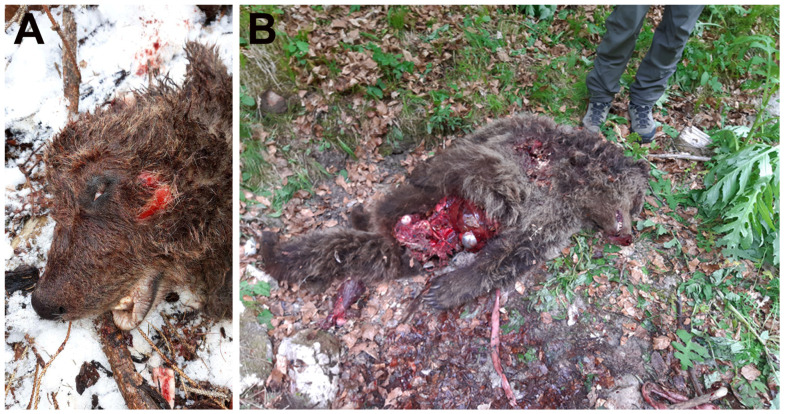
On-site inspection of the corpses of the yearlings. (**A**) Male M67, with cranial crushing and abrasions, recovered in Pler, Strembo (PAT, Italy). (**B**) Female F55, with abdominal lacerations and right pelvic limb amputation at the coxofemoral joint, recovered in Val d’Ambiez, San Lorenzo Dorsino (PAT, Italy).

The recovery sites where the corpses of the two yearlings were found yielded no substantial traces indicative of the aggressor (Figure 2).

**Figure 3:**
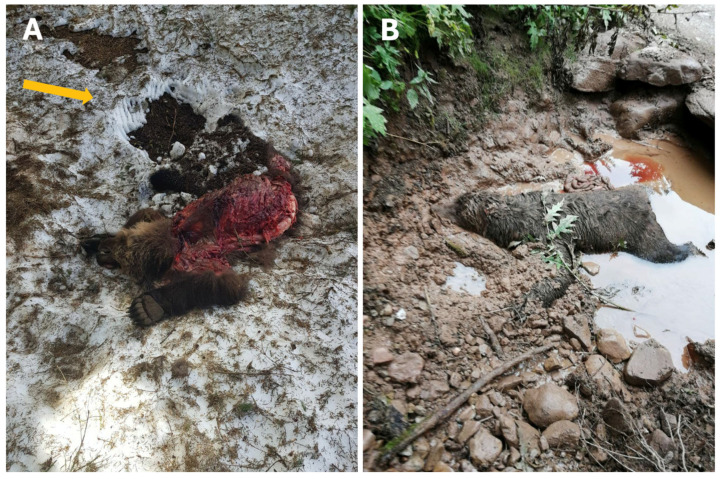
On-site inspection of the adult bears. (**A**) Male M71 exhibited extensive thoraco-abdominal lacerations involving both superficial and deep tissues. Unattributable claw marks, resulting from the agonistic interaction, were observed on the snow (orange arrow). The corpse was recovered in Viote del Bondone (PAT, Italy). (**B**) Male M50 was found in ventral recumbency in a muddy puddle at the edge of a forest road. Footprints were present but could not be conclusively attributed to any individual. The corpse was recovered in Cles Mountain (PAT, Italy).

The on-site survey at the recovery locations of adult bears revealed contrasting findings. At the M71 recovery site, claw marks were observed imprinted in the snow indicating that the final stages of the fighting likely took place at this site (Figure 3A). Claw markings and the snow presence allowed an estimation of the post-mortem interval, suggesting that the fight and subsequent death occurred after the recent snowfall, likely within two days prior to the corpse recovery.

In contrast, no reliable indicators on the M50 recovery site related to the fight were evident (Figure 3B).

### 3.3. Cadaveric Inspection and Lesion Study

#### 3.3.1. Diagnostic Imaging

**Figure 4:**
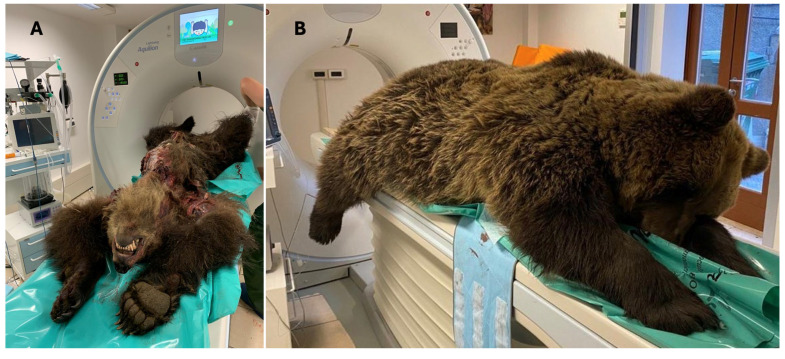
Computed tomography (CT) scan of the adult brown bears. (**A**) Scanning process of brown bear M71; (**B**) positioning of brown bear M50, highlighting its size in relation to the size of the instrument gate, which made it impossible to perform the examination.

CT investigations were only conducted on bear M71 because its size allowed it to pass through the instrument gate (Figure 4A). The two cases involving yearlings shared multiple and extended fractures of the skull bones and cervical spine. For this reason, CT scan evaluation was not considered useful for the study. The large size of brown bear M50 did not allow for the scan using the CT instrument (Figure 4B).

**Figure 5:**
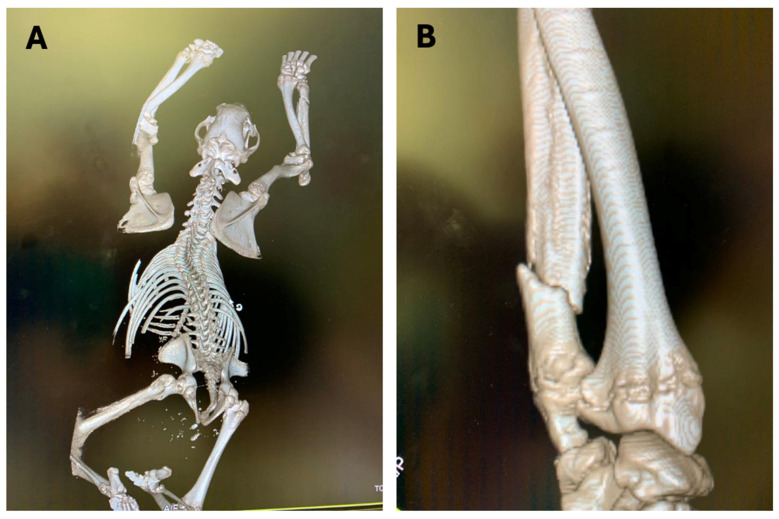
Computed tomography (CT) scan of brown bear M71. (**A**) Overall skeletal reconstruction, highlighting multiple fractures in the thoracic and pelvic regions; (**B**) displaced limb fracture, consistent with high-energy trauma.

Multiplanar and 3D reconstructions allowed a comprehensive assessment of the skeletal alignment, revealing multiple fractures involving the skull, the thoracic cage, pelvic girdle, and appendicular elements (Figure 5A), as well as a markedly displaced fracture of a limb (Figure 5B). These imaging findings are consistent with high-energy traumatic forces and corroborate the interpretation of biomechanical patterns underlying the aggression.

#### 3.3.2. General Cadaveric Inspection and Measurements

Macroscopic examination of the cavitary organs of the four brown bears subjected to autopsy revealed no signs of disease.

**Figure 6:**
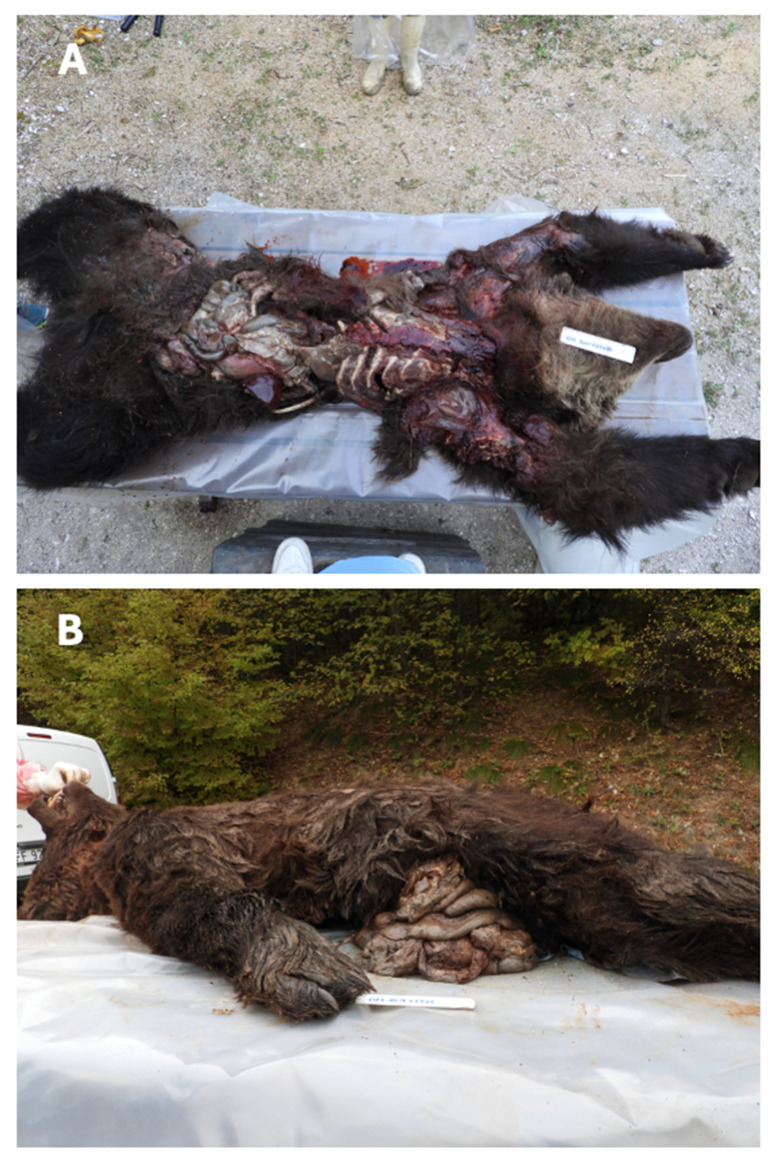
External cadaveric inspection. (**A**) Brown bear M71, showing extensive and widespread lacerations involving the thoracic, abdominal, and pelvic regions, as well as both thoracic and pelvic limbs. (**B**) Brown bear M50, displaying a large laceration in the medial and right abdominal regions, with exposure of intestinal loops. The figures provide an overview of the most significant lesions detectable through external examination of the corpses.

In all cases examined, extensive lacerations were evident in the regions of the skull, neck, ventral and lateral regions of the chest and abdomen, and inguinal regions, with exposure of the cavitary organs. Extensive lacerations were also observed in the medial regions of the limbs (Figure 6A,B).

**Figure 7:**
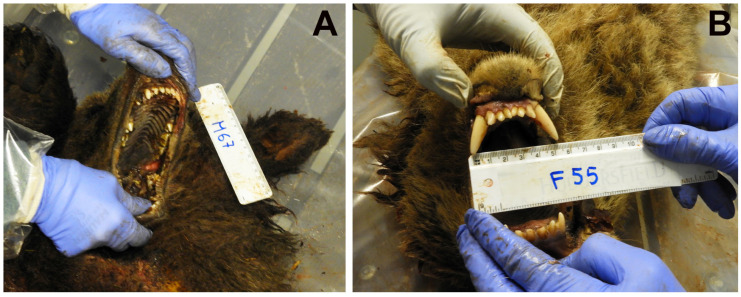
Inter-canine distance (I-CD) in the brown bear yearlings. (**A**) Male M67, with canine teeth in the initial eruption phase. Therefore, the I-CD was not measurable. (**B**) Female F55, exhibiting an I-CD of approximately 5 cm and a canine length of about 2.5 cm.

The yearling M67 showed facial deformation resulting from crushing trauma as evident in Figure 7A. Yearling F55 presented avulsion of the right pelvic limb. Bone fractures were perceptible on palpation.

The morphometric measurements of the brown bears were, in yearlings: male M67 aged 15 months, weighed 15 kg, measured 70 cm in length, and with a neck circumference of 32 cm; female F55 aged 16 months, weighed 25 kg, measured 80 cm from the occiput to the base of the tail, and with a neck circumference of 40 cm; in adults: male M71 aged 2 years and 4 months, weighed 88 kg, measured 98 cm from the occiput to the base of the tail, and with a neck circumference of 67 cm; male M50 aged 5 years and 8 months, weighed 180 kg, measured 133 cm in length, and with a neck circumference of 83 cm. The observed bear body size was consistent with the age and within the expected range of body mass and growth reported for brown bears in southern Europe [35].

The I-CD and canine length in the male yearling M67 was not measurable because the canines were in the eruption phase (Figure 7A); in the female yearling F55, the I-CD was approximately 5 cm and the canine length about 2.5 cm (Figure 7B).

**Figure 8:**
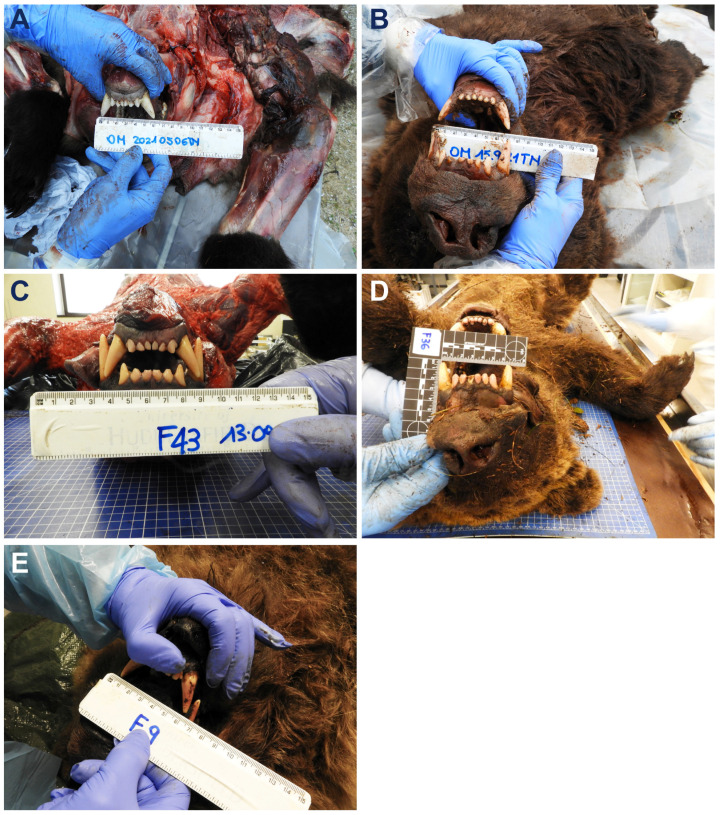
Inter-canine distance (I-CD) evaluation in the adult brown bears under study: male M71 (**A**) and male M50 (**B**), compared to females from the reference repository database F43 (**C**), F36 (**D**), and F9 (**E**) inhabiting the Italian Alps.

In the adult male M71, the I-CD was approximately 6 cm, with a canine length of about 3 cm, whereas, in the adult male M50, the I-CD was approximately 7 cm and the canine length was about 3.5 cm (Figure 8A,B). For comparison, I-CD measurements were also recorded in adult female brown bears (F43: 4 years and 8 months old, 130 kg, I-CD 5.5 cm; F36: 6 years and 8 months old, about 80 kg, I-CD 6 cm; F9: 8 years and 9 months old, 150 kg, I-CD 5.5 cm) from a reference dataset (Figure 8C–E). These individuals, who died respectively due to vehicular collision, gunshot injury, or in association with live-capture procedures, provide an important comparative context for the difference in I-CD according to sex and age in adult brown bears of the Italian Alps.

#### 3.3.3. Lacerations

Extensive traumatic lesions were documented in all cadavers examined. Lacerations varied in extent from a few centimeters to injuries involving entire anatomical regions, associated with hemorrhages due to vascular and parenchymal disruption.

Both yearlings exhibited multiple lacerations; in F55, a complete avulsion of the right pelvic limb distal to the hip joint was observed (Figure 2B).

Skin lacerations related to bite marks were observed on the nasal and frontal regions of bear M71 and on the frontal and parietal regions of bear M50 (see Section 3.3.4).

In the adult bears, the thoraco-abdominal diaphragm was torn, resulting in loss of intrapleural negative pressure and consequent pneumothorax. Hemothorax and pulmonary lacerations were also present. Intestinal loops were ruptured and partially exposed through breaches in the abdominal and inguinal walls (Figure 6A,B).

**Figure 9:**
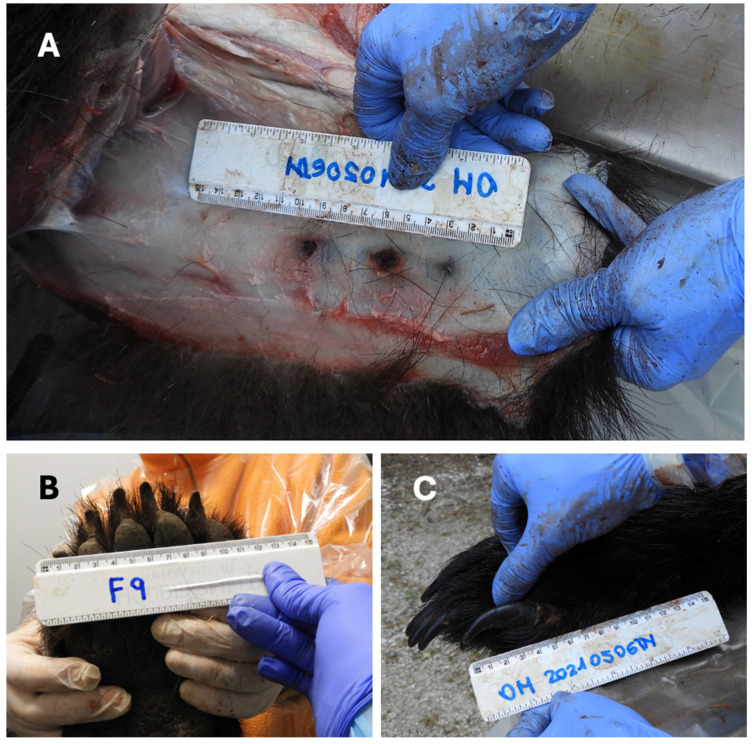
Measurements of claw-related lesions. (**A**) Bear M71, showing three aligned, circular lesions on the lateral region of the left thoracic limb, consistent with claw-induced trauma; (**B**) detail of the claw from adult bear F9 (subject deceased due to causes unrelated to the present study), provided to support interpretation of the lesion pattern observed in bear M71; (**C**) side view of the claw from adult bear M71.

Three circular lesions arranged linearly, ranging from superficial hematomas to perforating wounds with marked hemorrhagic halos, were identified in the subcutaneous tissue of the lateral region of the left thoracic limb of brown bear M71. The distances between perforations measured approximately 3.0–3.8 cm (Figure 9A,B).

#### 3.3.4. Bite Marks

No clearly identifiable bite marks were detected on the cadavers of the two brown bear yearling corpses.

**Figure 10:**
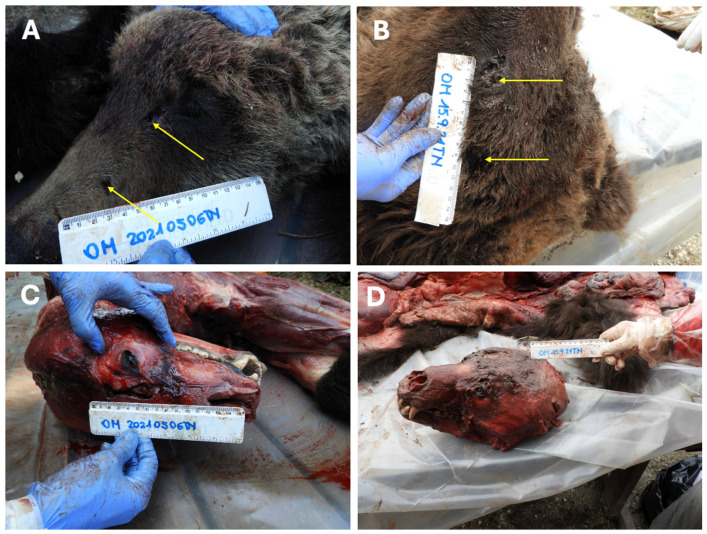
Bite marks and skull lesions in the adult brown bears. (**A**) Brown bear M71: skin penetrating holes spaced apart approximately 5 cm (yellow arrows). (**B**) Brown bear M50: measurable I-CD upon external examination of skin penetrating holes, spaced apart approximately 7 cm (yellow arrows) (frontal and parietal regions); (**C**) brown bear M71: skull fractures (nasal and frontal regions); (**D**) brown bear M50: no skull fractures.

Perforating skin wounds were documented in the cranial region of both adult brown bear corpses. Bite marks exhibited a circular shape with an approximate diameter of 6 mm, spaced apart about 5 cm in bear M71 and of about 7 cm in bear M50 (Figure 10A,B). Surrounding tissues showed minimal blood extravasation, indicating weak vital reactions.

In the perineal region of both adult brown bears, a laceration consistent with a bite was present, resulting in tissue avulsion. In M71, the penis and one testis had been avulsed. Due to deformation caused by tissue loss, I-CD measurement could not be performed. No blood extravasation was evident in perilesional tissues.

#### 3.3.5. Fractures

The autoptic examination confirmed the multiple fractures previously detected by CT scan (M71), and upon visual examination and palpation (yearlings), highlighting their dual nature: one type in the yearlings and another in the adult bears (Figure 2 and Figure 5).

Multiple crushing fractures were documented in both yearling cadavers (bears M67 and F55).

In the case involving the adult bear M71, extensive skeletal trauma was observed, including a skull fracture and multiple fractures of the ribs, pelvic bones, and both thoracic and pelvic limbs. The cranial fractures were consistent with a bite, affecting the nasal and frontal bones (Figure 10C). Rib and limb fractures were attributable to claw strikes (Figure 5).

In the adult bear M50, the only fracture detected during autoptic examination involved the floating ribs of the right costal region, also resulting from a claw strike. The bite to the skull in M50 produced a skin perforating lesion without associated bone fracture (Figure 10D).

#### 3.3.6. Genetic Analysis

Biological material collected from the labial papillae enabled genetic identification of the four brown bear cadavers as F55, M67, M71, and M50 and enabled us to perform age determination.

Conversely, genetic analysis of samples from perilesional tissues, teeth swabs, and distal phalanges of the adult brown bears M71 and M50 did not allow us to yield any DNA traces useful for identifying the conspecifics involved in the attack.

In the absence of clearly identifiable bite marks on the corpses of the two brown bear yearlings (F55 and M67), sample collection for detection of the genetic material of the attacker was not applicable.

#### 3.3.7. Macroscopic Analysis of the Gastric Content

Macroscopic examination of the gastric contents revealed that brown bear M71’s stomach contained some material consistent with a dairy product mixed with plant fibers. In the brown bear M50, a strong smoked odor was evident. The gastric content included pork rind measuring approximately 10 cm × 30 cm and numerous peppercorns, allowing the identification as remnants of speck, a typical regional cured meat product.

#### 3.3.8. Histological Analysis

**Figure 11:**
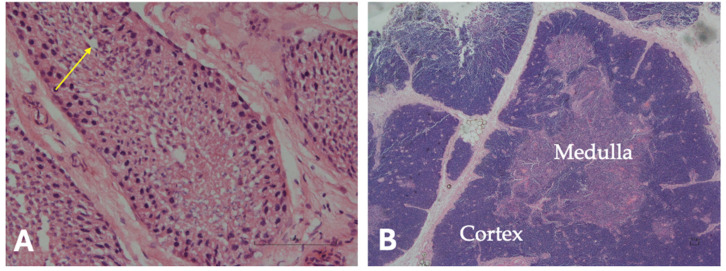
Histological investigation in the adult brown bear M71. (**A**) Testis, H&E stain, 20×: presence of spermatids within the seminiferous tubules (yellow arrow). (**B**) Thymus, H&E stain, 2×: thymus medulla and cortex are well represented, and no signs of atrophic involution with replacement by adipose tissue are observed. Scale bar = 100 µm.

Histological examination was performed on the testis and thymus collected from brown bear M71. Mild autolytic changes were observed. Autolysis prevented the evaluation of the seminiferous epithelium and Leydig cells. However, the testis showed elongated spermatids clustered near the lumen of the seminiferous tubules (Figure 11A). The thymus did not exhibit involution features (Figure 11B). Physiological involution of the thymus is characterized by atrophy of the medulla and the cortex, and by replacement with adipose tissue.

#### 3.3.9. Cause and Manner of Death

The codes used to identify the cause of death refer to the International Classification of Diseases 11th Revision (ICD-11), and are the following:

- Underlying causes: crushing injury of head (NA08) and cervical spine (NA22.3), traumatic pneumothorax (NB32.0), and multiple vascular injury (ND56.5);
- Immediate causes: hypoxia (MG44Y), traumatic shock (NF0A.4), and hypovolemic shock (MG40.1);
- Manner of death: assault by contact with animal “Assault by being crushed or stepped on by animal” (PE13) in relation with the mode of death of the yearlings and “Assault by being bitten by animal” (PE15) in relation with the mode of death of the two adult bears.

### 3.4. Behavioral Evidence

Behavioral analysis indicated distinct aggressive contexts. In the yearling cases, autopsy findings attributed the death to crushing trauma, characterized by diffuse bone fractures, severe internal lesions, and deep lacerations. This injury pattern is compatible with reasonable scientific certainty, with the manner of death classified as an assault by contact with animal (classified as “Assault by being crushed or stepped on by animal”) in the absence of evidence during on-site inspection and injuries compatible with other causes.

Conversely, in the adult brown bears, the predominant trauma, from a behavioral perspective, consisted of a forceful bite to the head; as reported before, in both cases this was accompanied by genital mutilation, and, in M71, with the avulsion of the penis and one testis. Gastric content analysis revealed the presence of anthropic-derived food items. These latter observations are suggestive of intraspecific aggression and conflict that could be driven by food or territory competition and possibly influenced by pheromone-mediated sexual signaling.

## 4. Discussion

This study examined the injury patterns associated with fatal intraspecific attacks among brown bears and analyzed the modalities of death in two yearlings and two adult individuals. In both groups, the lacerations observed were mainly in the ventral body regions and medial aspects of the thoracic and pelvic limbs. These injuries were attributable to the claw action. In addition, juvenile victims exhibited devastating traumas, including cranial crushing and multiple cervical spine fractures while, in both adult bears, a skull trauma due to a bite was observed. All lesions displayed vital signs, such as hemorrhages in the surrounding tissues. The study of the aspect of extravasation, which depends on the blood pressure in the vascular compartment, in terms of intensity and extension, allowed us to interpret the temporal sequence of the lesions: initial claw strikes followed by a crushing in the yearlings and a cranial bite in the adults.

This interpretation aligns with previous findings on perilesional blood effusions in a human cadaver victim of a bear attack [36], where claw wounds exhibited more pronounced hemorrhage than bite wounds. Extensive subcutaneous hemorrhage associated with claw injuries confirmed that these were inflicted ante-mortem, whereas minimal hemorrhages at bite sites suggests peri-mortem action following massive blood loss.

Severe hemorrhagic lacerations were mainly located in the thoracic limbs, thorax, abdomen and pelvis, indicating the initial phase of the attack. Their ventral and medial detection supports the hypothesis of an upright combat posture, consistent with reports that bears stand on the pelvic limbs during aggressive encounters [37]. This posture enables claw use to inflict major damage while maintaining cranial safety, which would not be guaranteed if the attack began with biting. Conversely, injuries with minimal or absent hemorrhage in the perineal regions reflect a post-mortem or agonic phase dominance behavior.

The identification of the aggressor relied on forensic odontology (bite mark analysis) and forensic genetics applied to suitable samples. Species determination from dental injuries is relatively straightforward unless marks are partial or distorted. The combined presence of claw-induced lacerations and clear bite marks excluded other sympatric apex predators. The literature indicates that fatal interspecific attacks among apex predators are rare [38]. For sex and age estimation, I-CD was assessed according to Murmann and co-workers [31], who provided extensive carnivore measurements.

Wolf predation typically involves total or partial consumption of the victim [39,40], except when interrupted or during surplus killing [41]. In the present cases, consumption was limited to perineal tissues, arguably linked to social dominance rather than feeding. Injury patterns, such as spacing of canine perforations and claw wound shape, cannot be confused with those inflicted respectively by wolves or lynxes.

A reliable I-CD measurement requires undistorted bite marks. Penetrating wounds observed at autopsy allowed an accurate assessment, excluding slippage or asymmetry that could compromise reliability. Distortion may occur due to (a) victim movement, (b) predator shaking and tissue avulsion, or (c) skin elasticity [42]. The victim movement at the time the bite was inflicted was negligible, given the attack sequence indicated by the hemorrhage aspect analysis. The bite force, influenced by the body masses ratio of those involved in the attack, plays a decisive role: brown bears exert approximately 2200 N of force [43], far exceeding the values reported for humans (120–350 N) [44]. Predators such as bears, whose feeding ecology includes large preys, have a high bite force [45].

If the load is applied slowly and gradually, the elastin fibers in the skin stretch and adapt to the load (elastic phase), whereas if a load is applied rapidly, like in the case of a bite from an attacking animal, the skin can break, and therefore tear, at lower stress levels [46]. Moreover, studies on human bites indicate minimal variation in the I-CD in different examined positions [47], due to limited canine crown width.

Based on the I-CD, aggressors were classified into two categories: (a) adult female or young male (<3 years), and (b) adult male.

Sexual dimorphism is a well-documented trait among mammals and, in brown bears, is closely linked to their social ecology. Adult males exhibit larger-in-width skulls compared to females, whereas skull morphology in individuals younger than 3 years does not allow reliable sex differentiation [5].

In the present study, as well as in comparative autopsy data, females and juveniles displayed a maximum I-CD of approximately 6 cm, while adult males exceeded this measurement (I-CD ≈ 7 cm). It is essential to apply a careful evaluation of the point-like lesions distinguishing a bite lesion from a claw lesion. Regarding the latter, claw spacing cannot be reliably assessed because digit position varies according to the muscular action during the attack.

Forensic odontology guidelines established by the American Board of Forensic Odontology (ABFO) recommend comparing bite mark evidence from the victim with dentition data from the suspect to enable inclusion or exclusion [48]. This process involves measuring I-CD and evaluating distinctive dental features such as anomalies or missing teeth. Applying these criteria to clearly identifiable bite marks, bear M71’s attacker was classified as an adult female or young male (I-CD ≈ 5 cm), whereas bear M50’s attacker was classified as an adult male (I-CD ≈ 7 cm).

Genetic identification represents a cornerstone of forensic investigations, provided that sampling, transport, and analysis adhere to internationally recognized standards and chain-of-custody protocols [49]. Forensic genetic testing must be conducted in accredited laboratories compliant with ISO/IEC 17011 [50] and EC Regulation 765/2008 [51]. Low-certainty identifications, where economic constraints influence methodological choices and interpretation relies on operator judgment, may be acceptable in wildlife management or compensation contexts, where species-level identification suffices [52,53]. However, such approaches are unsuitable for investigations aimed at identifying the actual aggressor.

The brown bear population in the Italian Alps originates from a limited founder group (3 males and 7 females), resulting in relatively low genetic diversity [52]. Most individuals in the Trentino region were genetically profiled through invasive (i.e., blood, labial papillae) and non-invasive (i.e., hair, feces) sampling. Hair samples are commonly collected from fur-rubbing sites such as trees, poles, or barriers, where bears rub for communication (pheromone marking), parasite control, or other behavioral purposes [23,54]. According to the investigative sciences, the proximity of hair or feces to a cadaver, or their presence on it, does not testify for temporal relevance, does not define the role in the action of the genetically identified subject and cannot reliably identify the aggressor [55] as it is evidence and not proof, by default [56].

Non-invasive molecular sexing poses no risk to animals or operators but may yield suboptimal DNA due to low quantity, degradation, or contamination [57]. Such limitations can lead to misclassification. Bidon and co-workers [58] proposed improved protocols to enhance reliability. Of fundamental importance is the integration between modern and traditional forensic techniques, such as forensic odontology applied in the present study.

In this study, peri-lesional samples were collected to recover traces of the attacker’s saliva. Limited to the two adult bears, perilesional samples were collected to recover traces of the attacker’s salivary DNA. The absence of clear bite marks on the bodies of the two yearlings made it inapplicable to identify the attacker by searching for salivary DNA. However, saliva in carnivores, composed of >99% water and minor organic components [59], is highly susceptible to degradation. The post-mortem interval (PMI), therefore, critically affects DNA integrity: research on wolf and dog predation in Italy demonstrated that, after 72 h of PMI, predator identification was impossible, and only 55% of samples collected within 36 h yielded species-level identification [60]. For this reason, we believe that it was not possible to detect DNA traces of the aggressor.

Finally, the seasonal context and sex of the aggressor may inform hypotheses regarding attack motivation. Macroscopic gastric content analysis revealed dairy residues in bear M71 and, in bear M50, residues of speck (regional cured meat). The trophic competition can occur in any period of bear activity [3,61]. Brown bear social organization is matrilinear [62], and individuals are generally solitary except during mating or maternal care. Bears exhibit high cognitive flexibility, curiosity, and memory, enabling adaptive behaviors in response to anthropogenic food sources [62]. Intraspecific and interspecific conflicts are primarily driven by offspring protection, reproductive success, and resource acquisition. Human activities often create food attractants, including wild animal foraging areas, hunting remains and farm animals, waste from households, industry, and tourism, which increase the likelihood of aggressive encounters [63].

The two yearling mortality cases, attributed to intraspecific aggression on the basis of the injury patterns and the exclusion of other sympatric predators, were not classified as SSI in the absence of aggressor identification.

Therefore, (a) the sex of the aggressor could not be determined; (b) the injury patterns are consistent with intraspecific aggression but do not allow attribution to a specific behavioral mechanism; and (c) SSI remains one possible but unconfirmed hypothesis, alongside other documented forms of conspecific attack.

In the cases of adulticide presented, the hypothesis that the aggression was triggered by competition for resources appears plausible.

## 5. Conclusions

To our knowledge, this study is the first to investigate the bear attack sequence based on the study of blood extravasation in perilesional tissues and to provide documentation and description of associated lesions (bites and claw wounds).

Gastric content analysis revealed anthropogenic food residues, dairy products in bear M71 and speck in bear M50 and highlighted that human-derived resources may have influenced the conflict. These findings provide two key insights: the attack modality and lesion characteristics, and the motivation behind aggression, which can extend beyond reproduction or maternal defense to include competition for food resources, as previously observed in other ursid populations.

From a management perspective, these results underscore the need to minimize anthropogenic attractants that increase bear–human interactions. Promoting coexistence through respect for ecological balance, aligned with “One-Health” and “Eco-Health” principles, is essential to prevent animal habituation to humans, which often leads to lethal outcomes for wildlife, such as poaching or the removal by managing authorities of individuals judged to be problematic or confidants.

Finally, this work opens perspectives for future research on the potential role of chemical communication in intraspecific conflicts in brown bears, a hypothesis that will require dedicated experimental investigations.

## Data Availability

The original contributions presented in this study are included in the article. Further inquiries can be directed to the corresponding authors.
